# Minor Antigen Disparities Impede Induction of Long Lasting Chimerism and Tolerance through Bone Marrow Transplantation with Costimulation Blockade

**DOI:** 10.1155/2016/8635721

**Published:** 2016-10-31

**Authors:** Sinda Bigenzahn, Ines Pree, Christoph Klaus, Nina Pilat, Benedikt Mahr, Elisabeth Schwaiger, Patrick Nierlich, Friedrich Wrba, Thomas Wekerle

**Affiliations:** ^1^Section of Transplantation Immunology, Department of Surgery, Medical University of Vienna, Waehringer Guertel 18, 1090 Vienna, Austria; ^2^Institute of Clinical Pathology, Medical University of Vienna, Waehringer Guertel 18, 1090 Vienna, Austria

## Abstract

Mixed chimerism and tolerance can be successfully induced in rodents through allogeneic bone marrow transplantation (BMT) with costimulation blockade (CB), but varying success rates have been reported with distinct models and protocols. We therefore investigated the impact of minor antigen disparities on the induction of mixed chimerism and tolerance. C57BL/6 (H2^b^) mice received nonmyeloablative total body irradiation (3 Gy), costimulation blockade (anti-CD40L mAb and CTLA4Ig), and 2 × 10^7^ bone marrow cells (BMC) from either of three donor strains: Balb/c (H2^d^) (MHC plus multiple minor histocompatibility antigen (mHAg) mismatched), B10.D2 (H2^d^) or B10.A (H2^a^) (both MHC mismatched, but mHAg matched). Macrochimerism was followed over time by flow cytometry and tolerance was tested by skin grafting. 20 of 21 recipients of B10.D2 BMC but only 13 of 18 of Balb/c BMC and 13 of 20 of B10.A BMC developed stable long-term multilineage chimerism (*p* < 0.05 for each donor strain versus B10.D2). Significantly superior donor skin graft survival was observed in successfully established long-term chimeras after mHAg matched BMT compared to mHAg mismatched BMT (*p* < 0.05). Both minor and major antigen disparities pose a substantial barrier for the induction of chimerism while the maintenance of tolerance after nonmyeloablative BMT and costimulation blockade is negatively influenced by minor antigen disparities.

## 1. Introduction

Donor specific tolerance through mixed chimerism can be achieved in various animal models by nonmyeloablative BMT and CB [1]. Success rates of chimerism and tolerance induction have typically been high when donor-recipient strain combinations were used which cross only MHC barriers but share the same mHAg background [2, 3]. However, this setting does not reflect the clinical situation where mHAg disparities exist universally. When switching to a different donor-recipient combination, which crosses MHC plus multiple mHAg barriers (Balb/c → BL6), retrospective review of pooled results revealed that overall only approximately 75% of mice developed lasting chimerism and of those that became chimeric approximately 15% rejected donor type skin grafts [4–7]. The potential impact of mHAg has not been formally tested, however.

mHAg are polymorphic non-MHC proteins that are able to induce a T cell response due to allelic variations between donor and recipient [8]. mHAg play a prominent role in hematopoietic stem cell transplantation (HSCT) in many regards [9]. Striking differences in engraftment of purified hematopoietic stem cells and/or development and severity of GVHD depending on mHAg disparities were found in murine MHC-matched donor host strain combinations [10]. In the human setting, disparities in mHAg increase not only the rates of rejection and graft-versus-host-disease (GVHD) [11] but also the efficacy of graft-versus-leukemia-effects (GVL) [12]. Recently, mHAg have also been reported to play an essential role in the persistence of donor chimerism [13].

mHAg also play a role in solid organ transplantation [14]. In mice, skin allografts differing only in minor antigens are rejected with the same pace as MHC disparate allografts [15]. Moreover, a single mHAg disparity was sufficient to induce chronic rejection of cardiac allografts in a congenic mouse model [16]. In clinical studies of kidney transplantation only limited and controversial data exist regarding the impact of mHAg disparities on graft survival [17]. Humoral immunity to specific mHAg, such as antibodies to angiotensin type 1 receptor (AT_1_R) and endothelin type A receptor (ET_A_R), have been shown to correlate with an increased incidence of acute rejection and inferior long-term graft survival in kidney and heart allografts [18].

However, so far the role of mHAg disparities in the induction and maintenance of tolerance through mixed chimerism has not been clearly defined. Therefore, we investigated different donor host strain combinations displaying MHC mismatches only (either H2^a^ or H2^d^) or MHC plus multiple mHAg mismatches (H2^d^).

## 2. Materials and Methods

### 2.1. Animals

Female C57BL/6 (BL6: H2^b^), Balb/c (H2^d^), and C3H/N (H2^k^) mice were obtained from Charles River Laboratories (Sulzfeld, Germany) and B10.D2 (H2^d^) and B10.A (H2^a^) from The Jackson Laboratory (Bar Harbor, ME). All mice were housed under specific pathogen free conditions and used between 8 and 10 weeks of age. All experiments were approved by the local review board of the Medical University of Vienna and were performed in accordance with national and international guidelines of laboratory animal care.

### 2.2. Conditioning Protocol and BMT

Age-matched female BL6 recipients underwent nonmyeloablative total body irradiation (TBI, 3 Gy, d − 1) prior to the intravenous injection of approximately 2 × 10^7^ unseparated bone marrow cells (BMC) from Balb/c, B10.D2, or B10.A donors as previously described [4–6]. Additionally mice were injected intraperitoneally with an anti-CD154 mAb (MR1; 1 mg d0) and hCTLA4Ig (0.5 mg d+2). Anti-CD154 mAb was purchased from Bioexpress Inc. (New Hampshire, USA) and hCTLA4Ig was generously provided by Bristol-Myers Squibb Pharmaceuticals (Princeton, New Jersey).

### 2.3. Skin Grafting

Full thickness tail skin from sacrificed Balb/c, B10.D2, or B10.A mice, respectively (donor specific), and C3H/N (H2^k^; 3rd party) was grafted 7 or 15 weeks after BMT. Recipient mice were anesthetized through intraperitoneal injection of a mixture of ketamine (100 mg/kg) and xylazine (5 mg/kg) before attachment of skin grafts at the lateral thoracic wall. Skin grafts were visually inspected thereafter at short intervals. Rejection was defined as less than 10% viable tissue.

### 2.4. Flow Cytometric (FCM) Analysis

Two-color FCM was used to distinguish donor and recipient cells of particular lineages, by staining with fluorescein isothiocyanate- (FITC-) conjugated antibodies against CD4, CD8, B220, and MAC1 and a biotinylated antibody against H-2D^d^ (34-2-12, developed with phycoerythrin streptavidin) and irrelevant isotype controls. To analyze the expression of V*β*-subunits staining was performed with FITC-conjugated antibodies against V*β*8.1/2 and V*β*11 and PE-conjugated antibodies against CD4. Propidium iodide (PI) staining was used to exclude dead cells. Mice were considered chimeric if they showed detectable donor cells within the myeloid lineage plus at least one lymphoid lineage. An Epics XL-MCL flow cytometer (Beckman Coulter, IL Alliance, Vienna, Austria) was used for acquisition and EXPO32 ADC Software, Applied Cytometry Systems, was used for analysis of flow cytometric data.

### 2.5. Histological Staining

Four micrometer sections were cut from paraffin-embedded tissue fixed in 4.5% formalin (pH of 7.5), stained with hematoxylin-eosin and Giemsa according to standard protocols, and analyzed by an experienced pathologist in blinded fashion according to The Banff 2007 Working Classification of Skin-Containing Composite Tissue Allograft Pathology [19].

### 2.6. Statistics

A two-tailed Student's* t-*test was used for comparing percentages of V*β*-positive populations and levels of chimerism within several cell lineages. The chi-square test was used for comparing rates of chimeras between groups. Skin graft survival was calculated according to the Kaplan-Meier product limit method and compared between groups by using the log-rank test. The Fisher Exact Test was used to compare histologically categorized skin grafts [19] of different donor groups. A *p* value less than 0.05 was considered to be statistically significant.

## 3. Results

### 3.1. Induction of Stable Multilineage Chimerism through BMT Plus CB Is Impeded by mHAg Disparities

**Figure 1:**
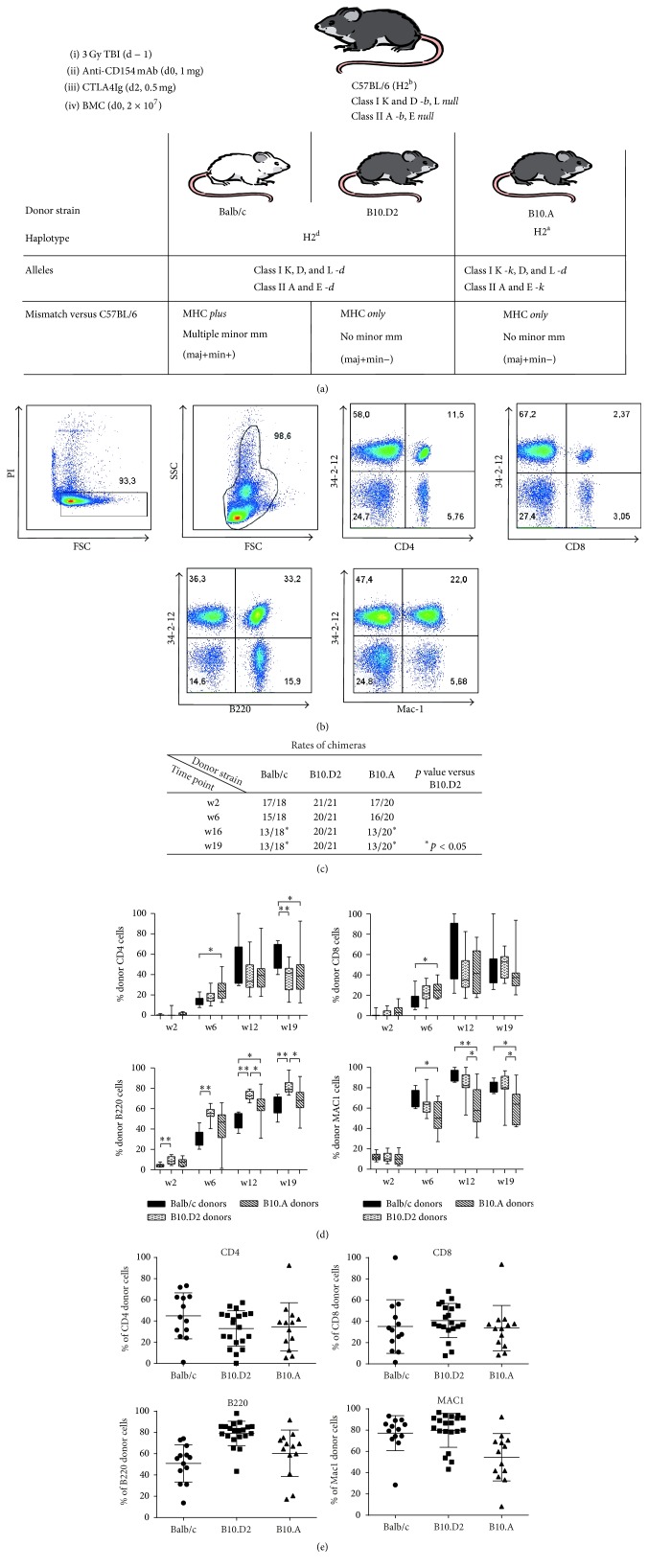
mHAg disparities impede rates of chimerism achieved through BMT with CB. (a) depicts the BMT protocol used with distinct donor-recipient combinations. (b) FACS plots show total donor (34-2-12 mAb recognizes H-Dd) and recipient type cells in CD4, CD8, B cells, and myeloid cells of one representative recipient of Balb/c BMC at the end of observation period. Dead cells were excluded through propidium iodide (PI) staining. (c) Rates of chimeras were determined at different time points after BMT. Early multilineage chimerism was induced in almost all mice. Maintenance of chimerism was observed in all but one recipient mouse of MHC mismatched, but mHAg matched B10.D2 (H2^d^) bone marrow throughout the observation period. In contrast, chimerism rates dropped in mice, which were transplanted with BMC of MHC and mHAg mismatched Balb/c (H2^d^) mice over time similarly to mice, which received MHC mismatched but mHAg matched B10.A (H2^a^) BMC (*p* < 0.05 for both versus B10.D2 BMC recipients after week 16). (d) Chimerism levels among CD4 cells, CD8 cells, B cells, and myeloid cells were measured by FCM at different time points after BMT. All groups of recipients showed relatively similar chimerism levels among T cell lineages throughout the observation period. Significantly higher chimerism was observed in B cells among recipients of MHC mismatched, but mHAg matched B10.D2 (H2^d^) bone marrow compared to mice transplanted with MHC and mHAg mismatched Balb/c (H2^d^) BMC (^*∗∗*^ *p* < 0.01 at all measured time points). Myeloid cell chimerism was significantly lower in recipients of MHC mismatched, but mHAg matched B10.A (H2^a^) after week 6 after BMT compared to recipients of Balb/c (H2^d^) BMC (^*∗*^ *p* < 0.05 and ^*∗∗*^ *p* < 0.01 at indicated time points). Mean percent of chimerism, interquartile range (box), and SD (whiskers) of long-term chimeras are shown as box-and-whisker blots (representative data from 1 of 2 independent experiments). (e) Chimerism levels of long-term chimeras after transplantation of Balb/c BMC, B10.D2 BMC, or B10.A BMC, respectively, at the end of observation period. Individual percent, mean percent of chimerism, and SD (error bars) are shown as scatter plot (pooled data of two independent experiments).

Three groups of mice were treated with a previously published nonmyeloablative BMT protocol [4–6]. BL6 recipients (H2^b^) received 2 × 10^7^ BMC from different donor strains with mismatches of MHC with/without additional mHAg mismatches after 3 Gy TBI (d − 1) together with CB consisting of a single dose each of anti-CD154mAb (1 mg MR1, d0) and CTLA4Ig (0.5 mg, d2). Balb/c (H2^d^), B10.D2 (H2^d^, same background as BL6), and B10.A (H2^a^, same background as BL6) mice were used as donors (Figure 1(a)) to investigate the potential influence of mHAg on top of the burden of MHC mismatch and the influence of the specific MHC haplotype on the induction of long-term multilineage chimerism (H2^a^ versus H2^d^). In BL6 recipients of Balb/c BMC high levels of multilineage chimerism (tested in CD4, CD8, B cells, and myeloid cells, Figure 1(b)) were initially induced in 17 of 18 mice. At 16 weeks after BMT only 13 of 18 mice stayed chimeric (pooled data of two independent experiments). This result is consistent with numerous previous experiments using this protocol showing that chimerism is lost in approximately 25% of recipients over time. In contrast, 21 of 21 BL6 mice receiving BMC of B10.D2 developed multilineage chimerism which remained stable in all but one animal over time (*p* < 0.05 versus Balb/c after week 16 after BMT, Figure 1(c)). Interestingly the rate of chimeras also dropped in BL6 recipientsof B10.A BMC from initially 17 of 20 immediately after BMT to 13 of 20 at week 16 (*p* < 0.05 versus B10.D2, Figure 1(c)). These data indicate that mHAg mismatches pose a barrier to establishing long lasting multilineage chimerism through BMT with CB. Additionally the MHC haplotype (H2^d^ versus H2^a^) also seems to influence bone marrow (BM) engraftment with this CB-based nonmyeloablative protocol.

Analyzing lineage-specific blood chimerism levels in successful long-term chimeras it was noted that B cell chimerism was significantly higher in recipients of B10.D2 BMC compared to recipients of Balb/c BMC at each investigated time point (e.g., B10.D2 versus Balb/c: 81.9% ± 7.2 versus 61.3% ± 9.4 at week 19, Figure 1(d), ^*∗∗*^ *p* < 0.01, ^*∗*^ *p* < 0.05), whereas CD8 and myeloid cell chimerism levels were comparable between these two groups. CD4 chimerism was significantly higher in recipients of Balb/c BMC at week 19 after BMT (B10.D2 versus Balb/c: 36.08% ± 14.53 versus 58.75% ± 12.13, Figure 1(d), ^*∗*^ *p* < 0.01). Significantly lower chimerism levels were observed in myeloid lineages in B10.A BMC recipients compared to recipients of Balb/c BMC from week 6 on. No consistent differences in T cell chimerism (CD4- and CD8 cells) and B cell chimerism levels were observed between long-term chimeras after BMT from B10.A or Balb/c donors. Nonetheless, CD4 and CD8 T cell chimerism levels were significantly higher (B10.A versus Balb/c: CD4: 25.56 ± 10.43 versus 13.51 ± 4.82 and CD8: 24.55 ± 7.83 versus 15.48 ± 8.86) in recipients of B10.A BMC 6 weeks after BMT but declined below that of Balb/c BMC recipients by week 19 (B10.A versus Balb/c: CD4: 37.71 ± 25.44 versus 58.75 ± 12.13, *p* < 0.05). Chimerism levels of individual BMT recipients are shown in Figure 1(e). The total level of donor chimerism among leukocytes was 62.41% versus 62.26% versus 53.41% in recipients of Balb/c versus B10.D2 versus B10.A bone marrow, respectively (at 19 weeks after BMT).

These results suggest that both individual MHC haplotype and minor antigen disparities influence the degree of chimerism in distinct lineages.

### 3.2. mHAg Disparities Are a Hurdle for Induction of Donor Specific Tolerance

**Figure 2:**
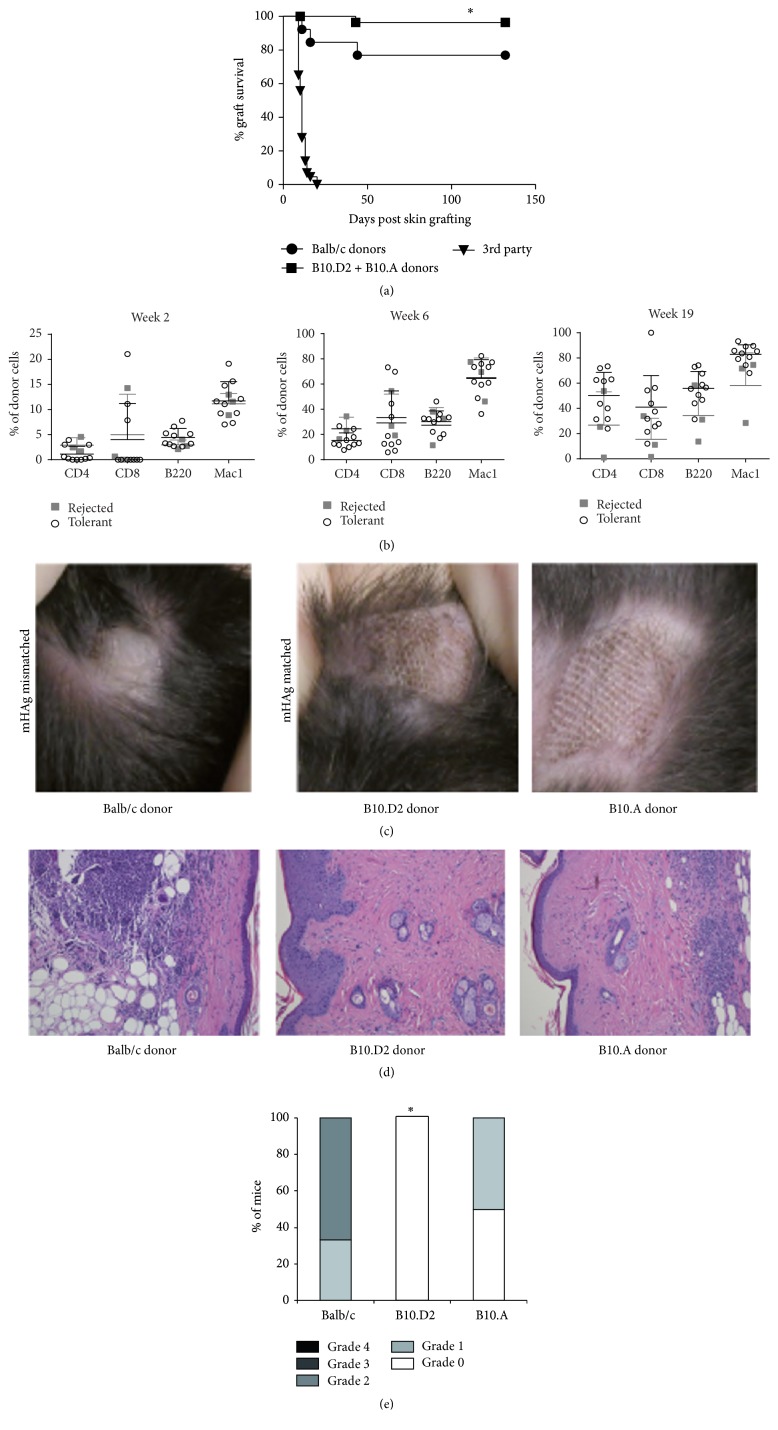
mHAg are a barrier for induction of donor specific tolerance through BMT plus CB. (a) BL6 (H2^b^) recipients of bone marrow of fully MHC mismatched Balb/c (H2^d^, mHAg mismatched), B10.D2 (H2^d^), or B10.A (H2^a^, both mHAg matched) mice were tested for donor specific tolerance through transplantation of donor type and 3rd party skin 7 or 14 weeks after BMT. Treatment with 3 Gy TBI (d − 1): transplantation of 2 × 10^7^ BMC together with CB (1 mg anti-CD154mAb, d0; 0.5 mg CTLA4Ig, d2) leads to long lasting (>130 days) donor skin graft acceptance in long-term chimeras, which had received BMC from MHC and mHAg mismatched Balb/c (H2^d^) donors in 10 of 13 mice. Significantly better graft survival was observed in long-term chimeric recipients of MHC mismatched but mHAg identical bone marrow (28/29, B10.D2 and B10.A, ^*∗*^ *p* < 0.05; pooled data from 2 independent experiments). Skin graft survival was calculated according to the Kaplan-Meier product limit method and compared by using the log-rank test. ^*∗*^ *p* < 0.05 versus Balb/c donors. (b) Chimerism levels of long-term chimeras, which received Balb/c BMC were not significantly different in tolerant mice (circles** ○**) compared to mice, which rejected (squares ■) donor skin grafts. *p* = n.s. for all time points. (c) Macroscopical aspects of Balb/c (H2^d^, mHAg mismatched) donor skin grafts changed during the observation period with grafts showing shrinking, thickening, and loss of surface structure. In comparison, donor grafts of B10.D2 (H2^d^) and B10.A (H2^a^, both mHAg matched) mice stayed macroscopically unchanged from 7 days after skin grafting (when protecting bandage was removed) until the end of observation period. (d) Representative histology of donor skin grafts of Balb/c (left), B10.D2 (middle), and B10.A (right) 44 weeks after skin grafting. HE staining, magnification 160x, and Giemsa staining 160x (not shown) analyzed. (e) Classification of donor skin grafts 44 weeks after skin grafting according to The Banff 2007 Working Classification of Skin-Containing Composite Tissue Allograft Pathology [19] by a blinded expert pathologist. ^*∗*^ *p* < 0.05 versus Balb/c. Most of Balb/c donor skin grafts showed histologically moderate (grade 2, 2/3) signs of inflammation whereas all B10.D2 donor skin grafts were free of inflammatory infiltrates (grade 0, *n* = 4, ^*∗*^ *p* < 0.05). B10.A partially showed no (grade 0, 2/4) or mild signs of inflammation (grade 1, 2/4, *p* = n.s. versus Balb/c).

Specific skin graft acceptance is considered as a stringent test to indicate transplantation tolerance. To investigate a possible influence of mHAg mismatches on skin graft acceptance, donor and 3rd party tail skin was transplanted 2-3 months after BMT. Among successfully established chimeras long-term donor skin graft survival (>130 days) was observed in 10 of 13 recipients of Balb/c BMC. This rate is similar to our previous experience with this protocol [4, 6, 7]. In contrast, recipients of B10.D2 or B10.A BMC showed a significantly better long-term donor skin graft survival (B10.D2: 16/17, B10.A: 12/12, Figure 2(a), ^*∗*^ *p* < 0.05 for Balb/c versus B10.D2 and B10.A donors; pooled data of two independent experiments). However, in recipients of Balb/c BMC no significant difference in chimerism levels of long-term chimeras, which rejected donor skin grafts in comparison to tolerant animals was observed (Figure 2(b)).

Regarding the macroscopic appearance of donor skin grafts of long-term chimeras, no shrinking, thickening, or loss of surface aspect was observed in mHAg matched B10.D2 and B10.A donor grafts in contrast to Balb/c grafts (Figure 2(b)). Histological analysis of those donor grafts that were retained until the end of follow-up revealed that Balb/c donor grafts exhibited signs of chronic rejection, like sparse infiltration with lymphocytes and mast cells together with focally dense lymphocytic-mononuclear cell infiltration. In comparison no incidence of dense focal lymphocytic infiltration was seen, in B10.D2 donor grafts (Figure 2(c)).

Skin grafts were scored by a blinded pathologist according to The Banff 2007 Working Classification of Skin-Containing Composite Tissue Allograft Pathology [19]. Moderate (grade 2; 2/3) and mild signs of inflammation (grade 1; 1/3) were found in Balb/c grafts, whereas no signs of inflammation (grade 0) were seen in 4/4 B10.D2 grafts (*p* < 0.05 versus Balb/c donors) and 2/4 B10.A grafts (2/4 grade 1, *p* = n.s. versus Balb/c, Figure 2(d)).

Taken together, these data suggest that mHAg disparities increase the rate of chronic skin graft rejection in recipients with persistent levels of mixed chimerism.

### 3.3. Deletion of Donor-Reactive T Cells Differs among Donor Strains

**Figure 3:**
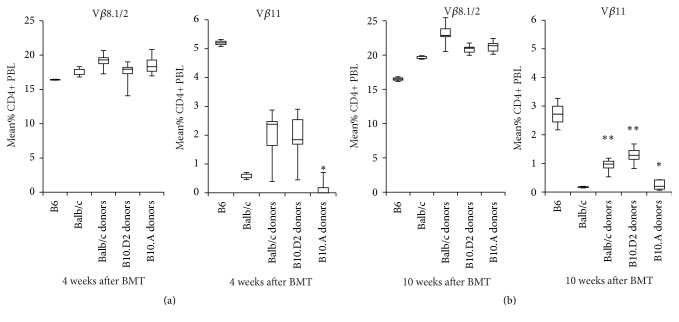
Deletion of donor-reactive T cells differs among donor strains. (a) Deletion of donor-reactive T cells was investigated through determination of the percentage of V*β*11^+^and V*β*8.1/2^+^CD4^+^PBL by 2-color flow cytometric analysis 4 weeks after BMT. Deletion of V*β*11^+^CD4^+^PBL in Balb/c (H2^d^, mHAg mismatched, *n* = 8) and B10.D2 (H2^d^, mHAg matched, *n* = 11) BMC recipients developed to a similar dimension irrespective of differing mHAg disparities of donor to recipient. In mice which were transplanted with BMC of B10.A (H2^a^, mHAg matched, *n* = 9) a significant increase of early deletion compared to BMT of Balb/c (H2^d^, mHAg mismatched) and B10.D2 (H2^d^, mHAg matched) donors was observed (^*∗*^ *p* < 0.05 versus Balb/c and B10.D2 BMC recipients). The percentage of V*β*8^+^CD4^+^ cells was not significantly reduced in any group compared to naïve BL6 mice indicating the specificity of the deletion for superantigens presented by the donor. (b) Ten weeks after BMT the degree of deletion was significantly enhanced in long time chimeras after Balb/c and B10.D2 BMT but still was significantly less pronounced than in recipients of B10.A BMC. Mean percentages of V*β*11^+^and V*β*8.1/2^+^CD4^+^PBL, interquartile range (box), and SD (whiskers) of long-term chimeras are shown as box-and-whisker blots. Representative data from one of two independent experiments. Statistical significance determined by log-rank test. ^*∗*^ *p* < 0.05 versus Balb/c donors; ^*∗∗*^ *p* < 0.01 versus week 4 after BMT (each group).

Peripheral and central clonal deletion are important mechanisms for the induction and maintenance of tolerance in models of mixed chimerism induced by BMT plus CB [20]. Notably not all mice become chimeric with such a protocol and not all chimeric mice accept donor skin grafts indefinitely. We followed V*β*11+ CD4+ T cells, which in this model recognize endogenous superantigens presented by donor MHC II (I-E) but not recipient MHC class II and thus serve as surrogate markers for donor-alloreactive T cells. Like in previous experiments Balb/c long-term chimeras displayed marked deletion of V*β*11+ CD4+ T cells by week 4 after BMT (2.04% ± 0.80 [*n* = 8] versus 5.20% ± 0.17 in naïve BL6 mice [*n* = 2]). Compared to Balb/c chimeras, a similar degree of deletion was observed in B10.D2 chimeras (1.99% ± 0.69 [*n* = 11], *p* = n.s.). Interestingly, transplantation of B10.A (H2^a^, mHAg matched) bone marrow led to a significantly more pronounced early deletion compared to Balb/c and B10.D2 (0.67% ± 1.62 [*n* = 9], *p* < 0.05 versus Balb/c and B10.D2 donors, both lineages). In contrast irrelevant V*β*8.1/2+ CD4+ T cells were not deleted in either group after BMT, indicating the specificity of the deletion for superantigens presented by the donor (Figure 3(a)). Ten weeks after BMT the degree of deletion was significantly enhanced in long time chimeras after Balb/c and B10.D2 BMT (0.93% ± 0.22 [*n* = 8] and 1.26% ± 0.27 [*n* = 11], *p* < 0.01 versus week 4 for both lineages) but still was significantly less pronounced than in recipients of B10.A BMC (0.23% ± 0.16 [*n* = 9], *p* < 0.01 versus Balb/c and B10.D2, both lineages; Figure 3(b)). Concluding, the type of MHC (i.e. I-E^d^ versus I-E^k^) may influence the extent and kinetic of deletion of donor-reactive T cells.

## 4. Discussion

In this study we provide evidence that mHAg disparities play a decisive role in the induction and maintenance of tolerance in recipients conditioned with nonmyeloablative BMT and CB, in which mHAg both impede the engraftment of BM and promote the rejection of donor skin in successfully established mixed chimeras.

Tolerance induction through donor BMT depends on two, mechanistically separate, events: successful engraftment of donor BM and durable tolerization of all relevant donor antigens in the context of donor chimerism. The findings presented herein indicate that mHAg influence both of these events. The frequency of successful long-term chimeras was higher in the absence of mHAg disparities when the MHC haplotype was the same (13/18 for Balb/c versus 20/21 for B10.D2). These results are in line with findings that MHC-matched BM is rejected in sublethally irradiated mice [21].

Only some of the minor antigens that drive alloreactivity in this strain combination (Balb/c to BL6) have been identified, with H60 provoking a particularly strong reaction [22]. H60 is a ligand for the activating receptor NKG2D which is expressed on NK cells and on activated CD8 T cells, which are both effective mediators of allogeneic BM rejection. Intriguingly, Balb/c donor mice feature a higher surface expression of H60 than recipient BL6 mice [23]. NKG2D has been reported to enhance NK cell mediated BM rejection in semiallogeneic pairs (Balb/c to F1) [24] but seems to be dispensable in fully mismatched combinations (Balb/c to BL6) [25]. Alternatively, NKG2D can function as a costimulatory receptor to augment the response of naïve CD8 T cells [26]. This could provide an alternative costimulatory route for CD8 T cells when CD40 and CD28 pathways are blocked. The role of NKG2D in CD8 T cell mediated BM rejection has not yet been evaluated although it is well established that H60 triggers an abnormally high number of responding CD8 T cells [22].

Unexpectedly, significantly fewer mice developed long lasting multilineage chimerism after receiving BM from B10.A compared to B10.D2 donors (both mHAg matched to recipient). Since the only difference between B10.A and B10.D2 is the H2 haplotype these results suggest that the MHC haplotype per se influences chimerism induction. Distinct H2 haplotypes are known to stimulate varying numbers of alloreactive T cells and thus exhibit a varying degree of immunogenicity [27], which apparently also influences BM rejection versus engraftment. Interestingly, if engraftment is successful, B10.A chimeras accepted donor skin grafts to a comparable extent as B10.D2 chimeras without macroscopical and histological signs of chronic inflammation. Thus, distinct MHC haplotypes impede BM engraftment to varying degrees but do not affect the success of skin graft tolerance in established mixed chimeras.

With regard to the second event, mHAg disparities increased the rate of skin graft rejection in successfully established chimeras (10/13 for Balb/c versus 28/29 for B10.D2 and B10.A). In addition, the surviving skin grafts of Balb/c but not B10.D2 donors exhibited histological signs of chronic inflammation. Thus, mHAg disparities can drive chronic rejection in the presence of stable mixed chimerism. Several groups attributed tissue specific antigens, which are not present in the BM but the skin, for this state of so-called “split tolerance” [28]. Obviously, for clinical translation this hurdle of mHAg disparities with its associated risk of “split tolerance” needs to be successfully overcome, which will require specifically designed protocols. Recently, we could demonstrate that the combination of regulatory cell therapy with donor BM transplantation leads to a state of tolerance that encompasses donor mHAg [7]. Critically, this regimen relies on extensive regulatory mechanisms, including linked suppression, that appear superior and indeed indispensable for tolerization of donor mHAg (Pilat et al. JCI Insight 2016, in press)

## 5. Conclusion

This study reveals that mHAg disparities have a negative impact on BM engraftment and tolerance maintenance in a nonmyeloablative, CB-based chimerism model. Preclinical tolerance protocols should encompass mHAg disparities to reflect the clinical setting and need to induce mechanisms capable of durable tolerization of donor mHAg.
